# MALDI-TOF MS Is an Effective Technique To Classify Specific Microbiota

**DOI:** 10.1128/spectrum.00307-23

**Published:** 2023-05-04

**Authors:** Liangqiang Chen, Wenjing Gao, Xue Tan, Ying Han, Fu Jiao, Bin Feng, Jinghang Xie, Bin Li, Huilin Zhao, Huabin Tu, Shaoning Yu, Li Wang

**Affiliations:** a Kweichow Moutai Group, Renhuai, Guizhou, People’s Republic of China; b Institute of Mass Spectrometry, School of Material Science and Chemical Engineering, Ningbo University, Ningbo, Zhejiang, People’s Republic of China; University of Maryland School of Medicine

**Keywords:** MALDI-TOF MS, microbiota classification, HCA

## Abstract

MALDI-TOF MS is well-recognized for single microbial identification and widely used in research and clinical fields due to its specificity, speed of analysis, and low cost of consumables. Multiple commercial platforms have been developed and approved by the U.S. Food and Drug Administration. Matrix-assisted laser desorption ionization–time-of-flight mass spectrometry (MALDI-TOF MS) has been used for microbial identification. However, microbes can present as a specific microbiota, and detection and classification remain a challenge. Here, we constructed several specific microbiotas and tried to classify them using MALDI-TOF MS. Different concentrations of nine bacterial strains (belonging to eight genera) constituted 20 specific microbiotas. Using MALDI-TOF MS, the overlap spectrum of each microbiota (MS spectra of nine bacterial strains with component percentages) could be classified by hierarchical clustering analysis (HCA). However, the real MS spectrum of a specific microbiota was different than that of the overlap spectrum of component bacteria. The MS spectra of specific microbiota showed excellent repeatability and were easier to classify by HCA, with an accuracy close to 90%. These results indicate that the widely used MALDI-TOF MS identification method for individual bacteria can be expanded to classification of microbiota.

**IMPORTANCE** MALDI-TOF MS can be used to classify specific model microbiota. The actual MS spectrum of the model microbiota was not a simple superposition of every single bacterium in a certain proportion but had a specific spectral fingerprint. The specificity of this fingerprint can enhance the accuracy of microbiota classification.

## INTRODUCTION

Mass spectrometry (MS) is a powerful analytical tool that ionizes compounds into charged molecules and measures their mass/charge ratio (*m/z*) (1). In 1996, Clayton et al., Holland et al., and Krishnamurthy et al. collected the peptide mass fingerprints of complete bacterial cells, opening the door to simple and rapid MS-based bacterial identification (2–4). Matrix-assisted laser desorption ionization–time-of-flight mass spectrometry (MALDI-TOF MS) technology has been continuously integrated and innovated with clinical diagnosis, food safety, environmental monitoring, and other fields in the rapid development process, ushering in a rapid detection revolution (5–8). MALDI-TOF MS has become the most mature and widely used method for single microbial identification, with the advantages of simplicity, accuracy, high throughput, and low cost compared to traditional culture-based biochemical testing (9).

In recent years, the developed MALDI-TOF commercial platforms include the Bruker (Bruker Daltonics, Germany) Microflex with the Biotyper database, the bioMérieux (Durham, NC) Vitek MS research-use-only system with the SARAMIS SuperSpectra database, the bioMérieux VitekMS *in vitro* diagnostic system with the Knowledge Base database, and Meihua (Zhuhai, China) M-Discover 100 mass spectrometer with its database (10, 11). Among these, MALDI Biotyper and Vitek MS have been approved by the U.S. Food and Drug Administration (12, 13). The identification principle of all of these platforms is mainly based on the fingerprint for bacterial ribosomal proteins in the m/z range of 2–20 kDa, which can be compared to the standard fingerprint in a database to identify the genus and species of bacteria (14, 15). Therefore, the database is the most critical component of the MALDI-TOF MS platform. However, the current various databases only report at the bacterial species level, and existing standard methods are only suitable for the analysis of single colonies purified by plate culture (16, 17). Microbes can present as a specific microbiota, and the detection and classification remain a challenge. Identification of these complex samples currently still requires plate culture to purify single colonies to obtain reliable results (18, 19). For example, MALDI-TOF combined with the method of microbial culturomics to study the diversity of gut microbiota has the advantages of being faster, more accurate, and costing less than the traditional method based on 16S RNA sequencing, and it is a reliable technique for exploring diversity (20, 21). However, the microorganisms contained in the microbiota are usually complex and diverse, and this method relying on separation and purification is cumbersome.

It is conceivable that MALDI-TOF is a more convenient method to directly characterize polymicrobial samples, but it is also a great challenge. Zhang et al. used a strategy based on similarity coefficients and biomarkers to identify bacteria in polymicrobial samples (22). Yang et al. developed a new framework to characterize polymicrobial samples without a purification procedure (23). Although these studies directly analyzed polymicrobial spectra, they still focused on how to identify single bacterium, ignoring the integrity. Changes in polymicrobial sample composition, especially the specific microbiota, are significant and usually indicate negative effects. For example, microbiota composition significantly influences chromaticity and the flavor of fermented food (24, 25), a lack of specific species of bacteria can lead to wilt in tomatoes (26, 27), and changes in gut microbiota composition can be used as biomarkers of obesity and type 2 diabetes (28). Therefore, detection of the overall difference between specific microbiota is a simpler method of analysis, which is conducive to food quality control, crop yield improvement, and disease treatment.

**FIG 1:**
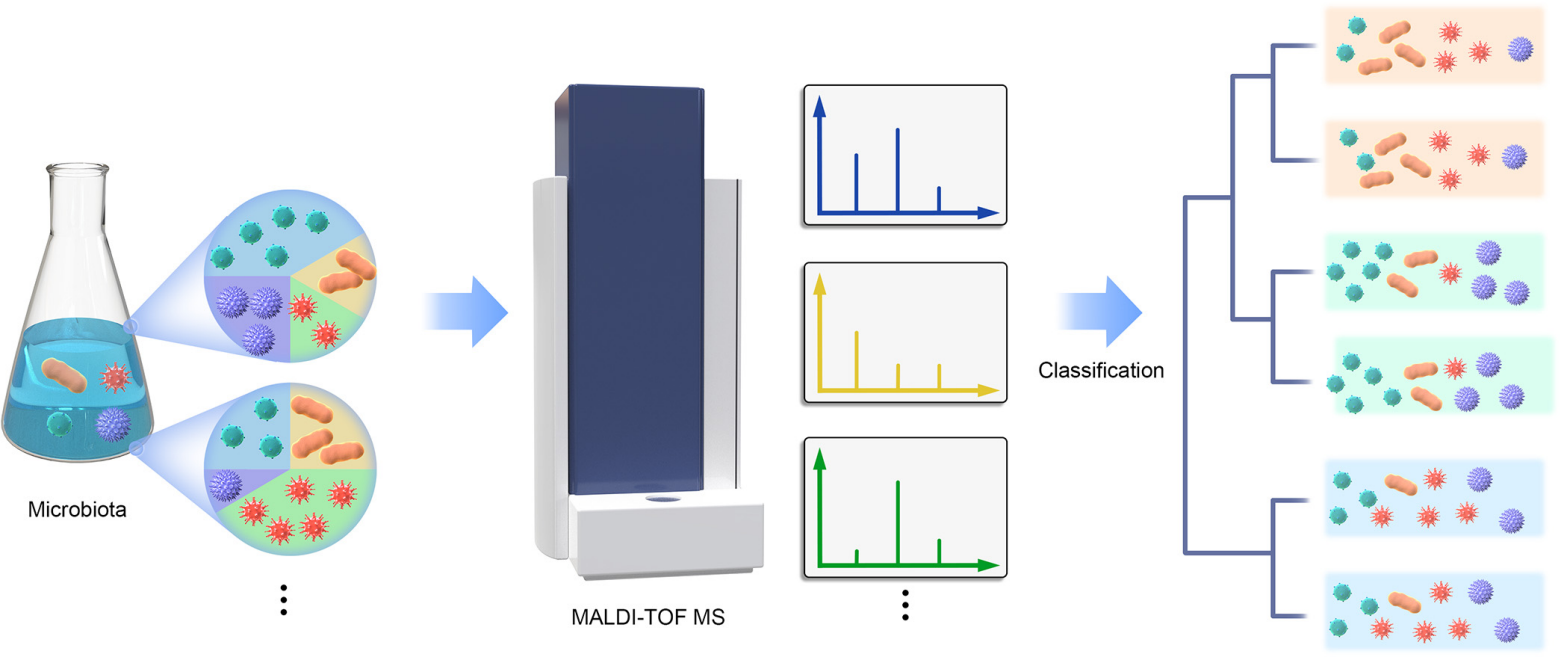
Workflow for classification microbiota based on MALDI-TOF MS.

Here, we proposed a new strategy to directly obtain the mass spectrum fingerprint by taking the microbiota as a whole. Microbiota classification was achieved by analyzing the differences in MALDI-TOF MS fingerprint spectra. To verify the feasibility of this method, we constructed 20 groups of specific model microbiota with different compositions of nine selected bacterial strains and classified them by MALDI-TOF MS (Fig. 1). The repeatability of MALDI-TOF MS for specific microbiota was investigated, and the actual and theoretical spectra of these microbiota groups were compared.

## RESULTS

### Overlap MS spectra of model microbiota.

Nine bacterial strains—namely, Klebsiella aerogenes, Citrobacter youngae, Staphylococcus aureus, Citrobacter freundii, Klebsiella pneumoniae, Bacillus cereus, Pseudomonas aeruginosa, Escherichia coli, and Acinetobacter baumannii—were selected to construct specific model microbiota. First, the MALDI-TOF MS spectra of these single bacteria were collected three times and identified using the MALDI-TOF MS database. Generally, the reliability of the spectrum was determined by the matching score. A score of >2.0 is considered reliable at the species level (29). As shown in Table S1 in the supplemental material, the matching scores of single bacteria were all >2.0, indicating that the collected mass spectra of single bacteria were of high quality. We accumulated three high-quality spectra for a single bacterium into a representative spectrum as shown in Fig. S1. Mass peaks are mainly distributed between 2 and 12 kDa. There were obvious differences in mass peaks between these single bacteria, which was also the basis of the MALDI-TOF MS identification of bacteria based on the differential expression of abundant bacterial ribosomal proteins (14, 15).

**FIG 2:**
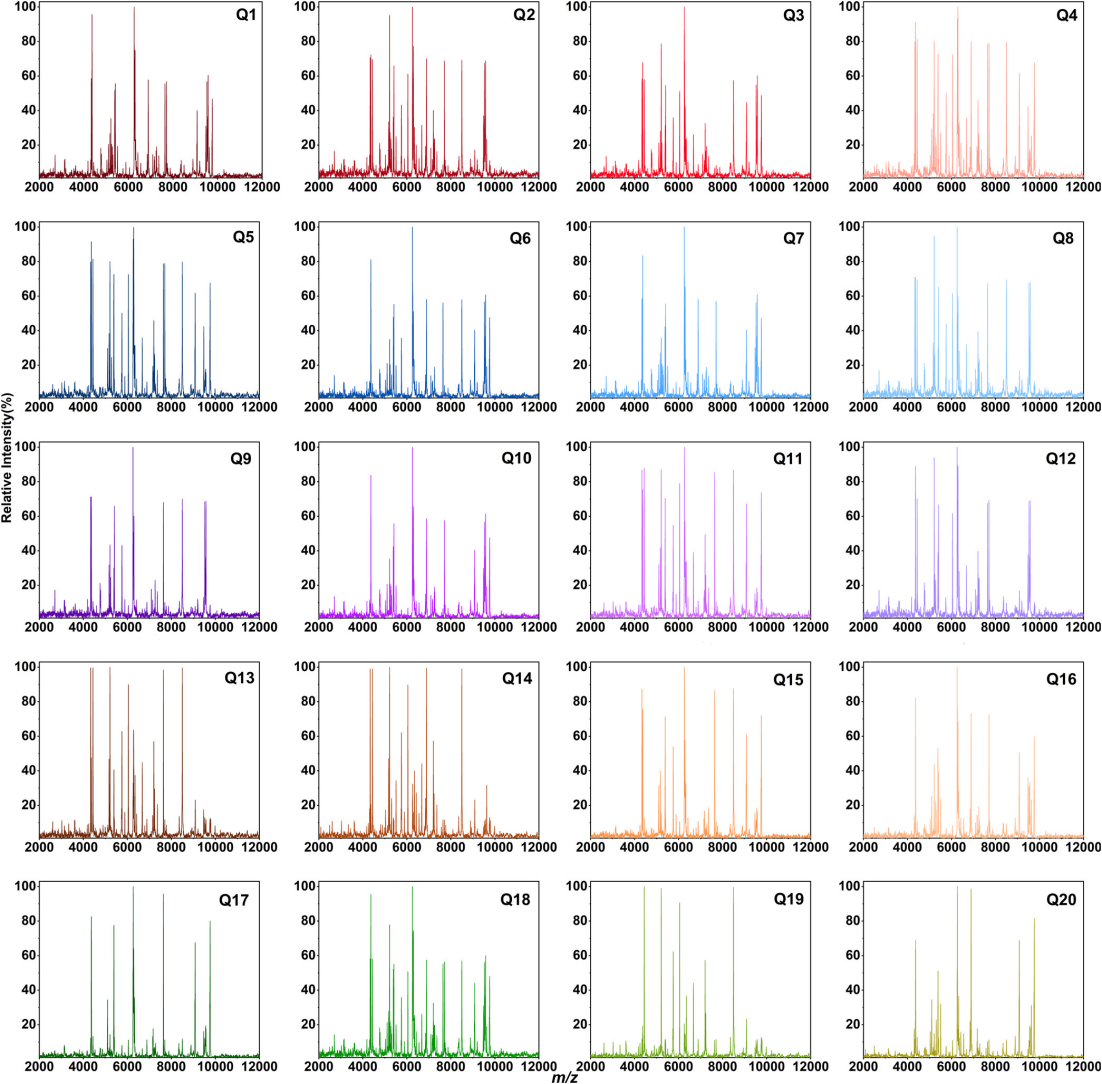
Overlap spectra of 20 specific model microbiotas.

**FIG 3:**
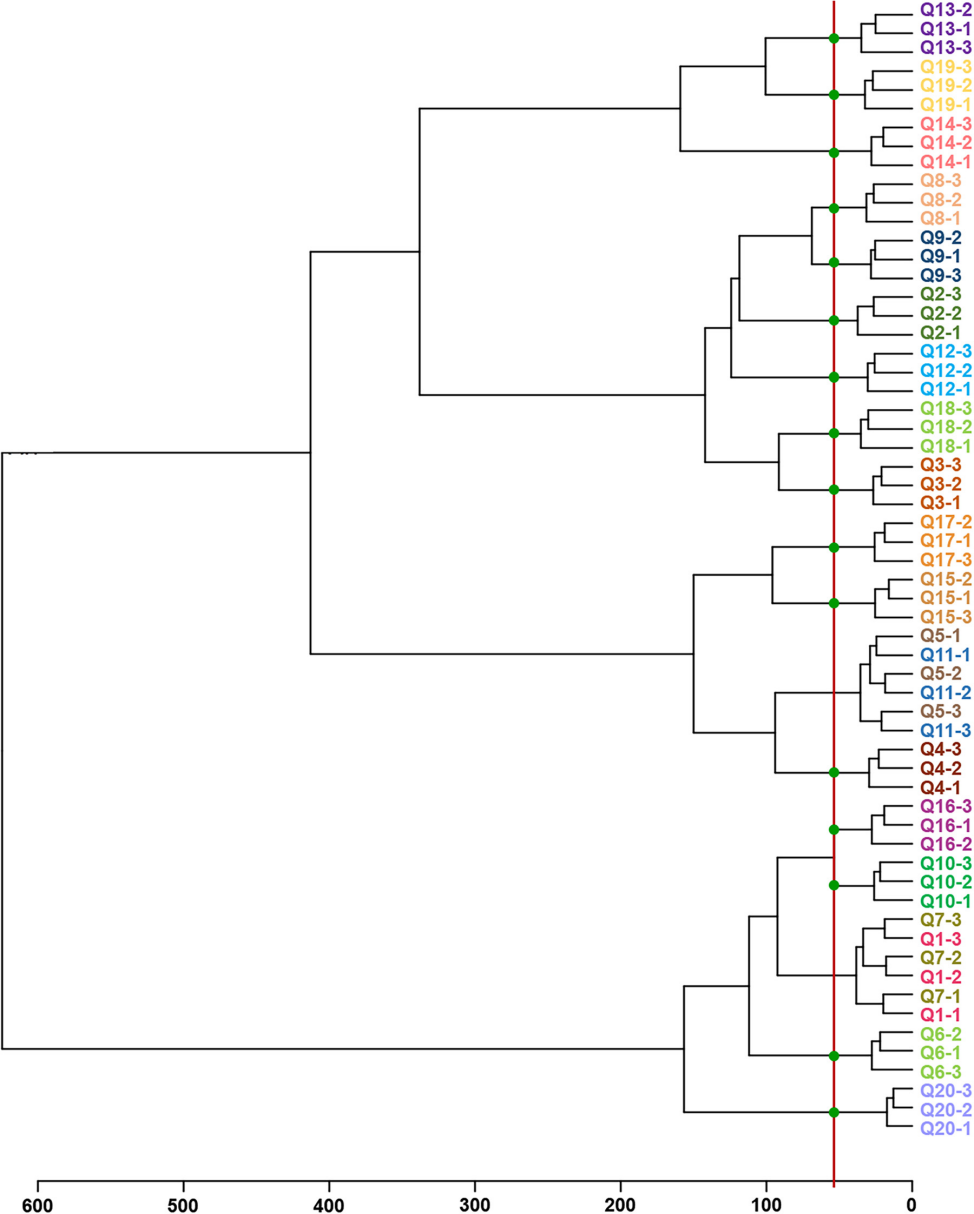
HCA-based classification of overlap spectra. The red line represents the threshold for model microbiota discrimination, and the green spots indicate that the model microbiota is clustered correctly. The abscissa represents the classification distance.

Theoretically, differences in the composition of the microbiota can also lead to differences in ribosomal proteins, resulting in different mass spectra. It is assumed that the model microbiota spectrum is the superposition of every single bacterial spectrum in a certain proportion. We verified whether the model microbiota superposition spectra could be classified by the commonly used hierarchical clustering analysis (HCA) method. According to the proportion of bacteria in Table S2, we weighted and overlapped the high-quality single bacterial spectra to obtain the overlap MS spectra of 20 model microbiotas. Figure 2 shows the 20 groups of overlap MS spectra with abundant mass spectral peaks. The overlap MS spectrum of each microbiota could be classified by HCA, with an accuracy of 80% (Fig. 3). The overlap spectra of the model microbiota with similar proportions were close together. For example, the proportions of Q1 and Q7 were similar. Only the content of Klebsiella aerogenes was different. Their spectra were similar and misclassified into the same group. Q1 and Q19 were groups with completely different proportions, and their overlap spectra were clustered far away. These results showed that MALDI-TOF MS can classify the overlap spectra of specific microbiota, and the greater the composition difference of specific microbiota, the better the classification effect.

### Reproducibility of actual MS spectra of model microbiota.

The nine selected bacteria were cultured overnight in liquid Luria-Bertani (LB) medium, and the concentration of each single bacterium was measured by plate count agar (see Table S1). A total of 20 specific model microbiotas were constructed according to the volumetric proportions shown in Table S2, and the mass spectra were collected. Reproducibility is important for determining reliable results using MALDI-TOF MS (30). In order to ensure stability and representativeness, sample preparation was repeated five times. Figure S5 shows five repeated spectra for each model microbiota. We observed that the mass spectra exhibited similar profiles between each run, indicating excellent reproducibility and stability of the MS-based method.

### Comparison of actual microbiota MS spectra with overlap MS spectra of single strains.

The actual collected spectra and overlap spectra are shown in Fig. S6. The similarity between the actual spectra and the overlap spectra is still determined by the matching score. A score >2.0 is considered an exact match. A total of 70% of the spectral matching scores were < 2.0, which means that the actual MS spectrum of the model microbiota was different than that of the overlap spectrum. This result suggests that the actual MS spectrum was not a simple superposition of every single bacterium in a certain proportion but had a unique fingerprint. Overlap MS spectra were obtained by superposition of all single bacterial spectra. Therefore, all peaks in the single bacterial spectra can be observed in the overlap MS spectra (see Fig. S6, blue). However, the MS peaks abundance of the actual MS spectra (see Fig. S6, red) decreases obviously. This is reasonable because different bacteria differ in the amount of protein and the efficiency of MALDI-TOF detection (23). The MS signals of different bacteria may interfere with each other during simultaneous detection, resulting in a decrease in the abundance of MS peaks. Our previous research also found that characteristic peaks of higher intensity have a suppression effect on other peaks (31). These factors work together to enhance the specificity of the microbiota fingerprint.

**FIG 4:**
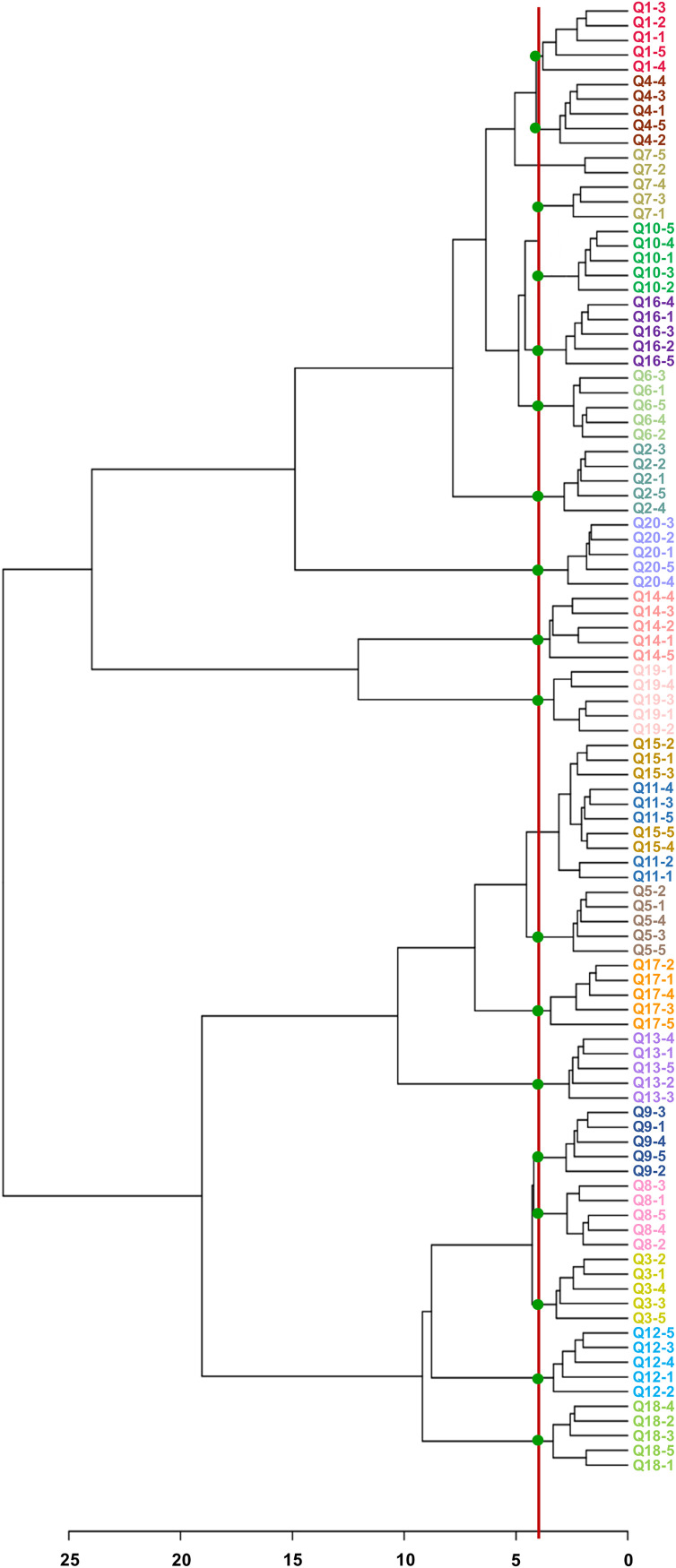
HCA-based classification of actual MS spectra. The red line represents the threshold for model microbiota discrimination, and the green spot indicates that the model microbiota is clustered correctly. The abscissa represents the classification distance.

The actual MS spectra of specific model microbiota showed excellent repeatability and were easier to classify by HCA. As shown in Fig. 4, 17 sample groups were correctly clustered (Q7, Q11, and Q15 were not clustered appropriately), and precise typing result was obtained with an accuracy of 85% (Fig. 4), which is very intuitive in principal component analysis results (shown in Fig. S2). At the same time, the actual MS spectra of model microbiota with similar proportions were close by clustering. This was similar to the overlap MS spectra. However, the clustering groups of the actual fingerprint spectra were obviously different from the overlap fingerprint. This result reflects the specificity of the actual collected model microbiota fingerprint spectra. This specificity of the fingerprint can enhance the accuracy of classification.

## DISCUSSION

Traditional microbial studies rely on culturing and phenotypic characterization, which is time-consuming and laborious due to the long culture cycle (32). Moreover, many microorganisms are not culturable, significantly reducing the diversity of the microbiota for further study. Analytical methods based on direct nucleic acid extraction can overcome these limitations, including PCR-denaturing gradient gel electrophoresis, time-temperature-gradient gel electrophoresis, and quantitative real-time PCR (24). These approaches are widely used for identifying the microbial composition of microbiota and allow broad characterization of microbial diversity (33–36). However, the technology requires complicated steps and is expensive. With the rapid development of high-throughput sequencing technology, gene sequencing has become a sharp tool for studying microbiota. Recently, methods such as amplicon sequencing and metagenomic sequencing were developed. Amplicon sequencing (16S rRNA, 18S rRNA, and internal transcribed spacer sequencing) is a highly targeted method used to study microbiota composition, diversity, and evolutionary relationships between species (37, 38). Metagenomic sequencing provides more information and can be further studied after species annotation is completed (39, 40). However, sequencing-based methods are more expensive and require professionals to manipulate and analyze the data. These existing methods use detailed analysis of the information on the species contained in the microbiota to determine the differences between different microbiota. These continuously developing research methods are gradually unveiling the mystery of microbiota. However, it is undeniable that these methods are often used in laboratory studies and are not timely guidance for practical applications, such as clinical diagnosis or industrial production. In the present study, we propose a new strategy to directly obtain the mass spectrum fingerprint by taking the microbiota as a whole. Microbiota classification was achieved by analyzing the differences in MALDI-TOF MS fingerprint spectra. Compared to the methods described above, the proposed method is a means of general distinction, which is fast, simple, inexpensive, and more suitable for application in real-world scenarios. For example, during the production of Baijiu, the quality of Daqu is closely related to the microbiota composition. We think this method can quickly classify Daqu of varying quality and be applied to the production process.

The results show that MALDI-TOF MS is an effective technique for classifying specific microbiota. The specific microbiota spectra collected by MALDI-TOF MS showed excellent repeatability and could be classified by the commonly used HCA with high accuracy. The actual MALDI-TOF MS spectrum of a specific microbiota was not a simple superposition of every single bacterium in a certain proportion but had a specific fingerprint with high repeatability. These results indicate that the widely used MALDI-TOF MS identification method for individual bacteria can be expanded to microbiota. Some challenges remain for practical application. In actual sample analysis, there is interference from many impurities, demanding specific and effective pretreatment methods according to the specific characteristics of different samples. In addition, microbiota possess vigorous bioactivity with abundant metabolites, such as enzymes and other biomacromolecules. The mass signals (*m/z* range of 2 to 20 kDa) of these substances will mix with the signal of the whole microbiome and affect the classification accuracy. If the metabolites are different, it may enhance the accuracy of the classification to some extent. Subsequent research on actual samples should be conducted for verification.

## MATERIALS AND METHODS

### Reagents and equipment.

Centrifuge tubes, masks, and safety glasses were purchased from Shanghai Titan Scientific Co., Ltd. (Shanghai, China). α-Cyano-4-hydroxycinnamic acid (CHCA), formic acid (analytical reagent [AR]), anhydrous ethanol (AR), trifluoroacetic acid (AR), and acetonitrile (ACN) were purchased from Merck (Darmstadt, Germany). Tryptone soybean agar and LB broth were purchased from Beijing Land Bridge Technology Co., Ltd. (Beijing, China). An ATL-032R digital shaker (Shanghai Chemstar Instruments Co., Ltd. China), centrifuge (Shanghai Boyu Instruments Co., Ltd.), and autoclave (Shanghai Boxun Industry & Commerce Co., Ltd.) were used. The MALDI-TOF MS system used an M-Discover 100 mass spectrometer (Zhuhai Meihua Medical Technology Co., Ltd., China) in linear positive mode with *m/z* range 2 to 20 kDa. Metabo Analyst 4.0 (McGill University, Montreal, Canada) was used for analysis (41).

### Sample preparation.

All selected bacterial strains were obtained from China Center of Industrial Culture Collection (CICC) and American Type Culture Collection (ATCC). The nine strains were Escherichia coli (ATCC 8739), Klebsiella aerogenes (ATCC 13048), Citrobacter youngae (ATCC 29935), Staphylococcus aureus (ATCC 29213), Acinetobacter baumannii (ATCC 17978), Citrobacter freundii (CICC 10404), Klebsiella pneumoniae (CICC 21106), Bacillus cereus (CICC 10041), and Pseudomonas aeruginosa (CICC 21636).

Nine strains were cultured in 20 mL of liquid LB medium at 37°C for 14 h with consecutive shaking at 175 rpm. After we removed the LB broth by centrifugation at 12,000 rpm for 5 min at room temperature, we washed the strains three times with sterile water. The purpose of the washing step is to remove salt and impurities in the culture solution to avoid affecting the subsequent mass spectrum signal collection. The concentration of each single bacterium was measured by plate count agar as shown in Table S1.

The 20 model microbiota samples were constructed according to the proportion shown in Table S2 for subsequent MALDI-TOF MS detection. The 20 model microbiota samples were named Q1-Q20. The proportion was designed systematically. The dominant bacteria in each sample were different, with Q1 to Q4 dominated by seven bacteria, Q5 to Q8 dominated by six bacteria, Q9 to Q12 dominated by five bacteria, Q13 to Q16 dominated by four bacteria, and Q17, Q19, and Q20 dominated by three bacteria. The proportion of bacteria in Q18 was the same. The purpose of this design was to make the proportions as rich as possible to verify the classification ability of MALDI-TOF MS. Figure S4 shows the composition of the microbiota visually.

### MALDI-TOF MS spectra acquisition.

The on-plate extraction standard method and CHCA matrix were pretreated for single bacterial samples and model microbiota samples (17). On-plate extraction refers to protein extraction on the target plate. The specific steps are as follows: (i) apply the sample points to the target plate and dry; (ii) mix with 0.5 μL of 70% formic acid, followed by 0.5 μL CAN; (iii) dry at room temperature; and (iv) cover the CHCA matrix solution. Five parallel mass spectra were acquired for each sample with a 337-nm N_2_ laser in linear positive mode with a mass range of *m/z* 2 to 20 kDa. Each spectrum was acquired by a sum of 300 shots in increments of 60, and the laser intensity was regulated to ensure a good signal-to-noise ratio. The instrument was calibrated using the quality control bacteria (Escherichia coli ATCC 8739) according to the manufacturer’s instructions. When identification scores for the quality control bacteria were >2.0 in MicroCtrl 1.0, new spectra were acquired in manual acquisition mode. According to the manufacturer, a score of <1.7 indicated an unreliable result, a score of 1.7 to 2.0 indicated generic identification, and a score of >2.0 was considered accurate identification at the species level.

### Data preprocessing and analysis.

The collected MALDI-TOF MS data for single bacterial samples and model microbiota samples were preprocessed, including a series of processing steps, such as smoothing, baseline correction, and normalization. The detailed processing steps are described in our previous study (15). Briefly, the obtained data were imported into MetaboAnalyst 4.0 to perform HCA. Euclidean distance and Ward’s algorithm were applied for HCA analysis (15). If the repetition spectra of a sample clustered into a group under the heterogeneity threshold (red line in the HCA classification), the sample was considered to be correctly classified. Classification accuracy was defined as the number of samples correctly classified divided by the total number of samples (total sample number, *n* = 20).

### Preparation of model microbiota overlap spectra.

The overlap spectrum was obtained to integrate the mass spectrum peaks of all single bacteria into one spectrum. The operation was as follows. First, a standard spectrum was obtained for each single bacterium. The standard spectrum needs to be representative, so each was the accumulation of three repeated spectra, which was directly completed in the MicroCtrl 1.0 software. The standard spectral data for each single bacterium were then imported into Origin software for normalization. The overlap spectra were constructed according to the proportions in Table S2; that is, the mass spectrum peak of model microbiota was equal to the mass spectrum peak intensity of a single bacterium multiplied by the corresponding percentage coefficient and then added. It can be expressed by the following formula (taking Q1, for example):

$$\begin{array}{l} \text{Q1}=\mathrm{Ec}\times 17.86\%+\mathrm{Bc}\times 8.24\%+\mathrm{Sa}\times 10.40\%+\mathrm{Kp}\times 13.34\%+\\ Cf\times 16.29\%+\mathrm{Ab}\times 1.59\%+\mathrm{Pa}\times 1.47\%+\mathrm{Cy}\times 15.89\%+\mathrm{Ka}\times 14.91\% \end{array}$$

where *Ec* = Escherichia coli, *Bc* = Bacillus cereus, *Sa* = Staphylococcus aureus, *Kp* = Klebsiella pneumoniae, *Cf* = Citrobacter freundii, *Ab* = Acinetobacter baumannii, *Pa* = Pseudomonas aeruginosa, *Cy* = Citrobacter youngae, and *Ka* = Klebsiella aerogenes.
