# Supplementary material for: MALDI-TOF MS Is an Effective Technique To Classify Specific Microbiota
Source: Microbiol Spectr. 2023 May 4;11(3):e00307-23. doi: 10.1128/spectrum.00307-23 (PMC10269913; doi:10.1128/spectrum.00307-23)
Supplement: Supplemental file 1 — Supplemental material. Download spectrum.00307-23-s0001.docx, DOCX file, 1.8 MB [file spectrum.00307-23-s0001.docx]

**Supporting Information**

**MALDI-TOF MS is an Effective Technique to Classify Specific Microbiota**

*Xue Tan^a, 1^, Wenjing Gao^b, 1^, Ying Han^a^, Fu Jiao^a^, Bin Feng^b^, Jinghang Xie^b^, Bin Li^b^, Huilin Zhao^b,2^, Huabin Tu^a^, Shaoning Yu^b,^***, and Li Wang^a,^**

^a^ Kweichow Moutai Group, Renhuai, Guizhou 564501, China

^b^ Institute of Mass Spectrometry, School of Material Science and Chemical Engineering, Ningbo University, Ningbo, Zhejiang, 315211, China

*1,* These authors contributed equally to this work. *2*, Summer student from Shanghai Xingzhi high School.

***Corresponding Author:**

Li Wang, Kweichow Moutai Group, Renhuai, Guizhou 564501, China. Electronic address: wanglimoutai2021@163.com;

Shaoning Yu, Institute of Mass Spectrometry, School of Material Science and Chemical Engineering, Ningbo University, Ningbo, Zhejiang, 315211, China. Electronic address: yushaoning@nbu.edu.cn.


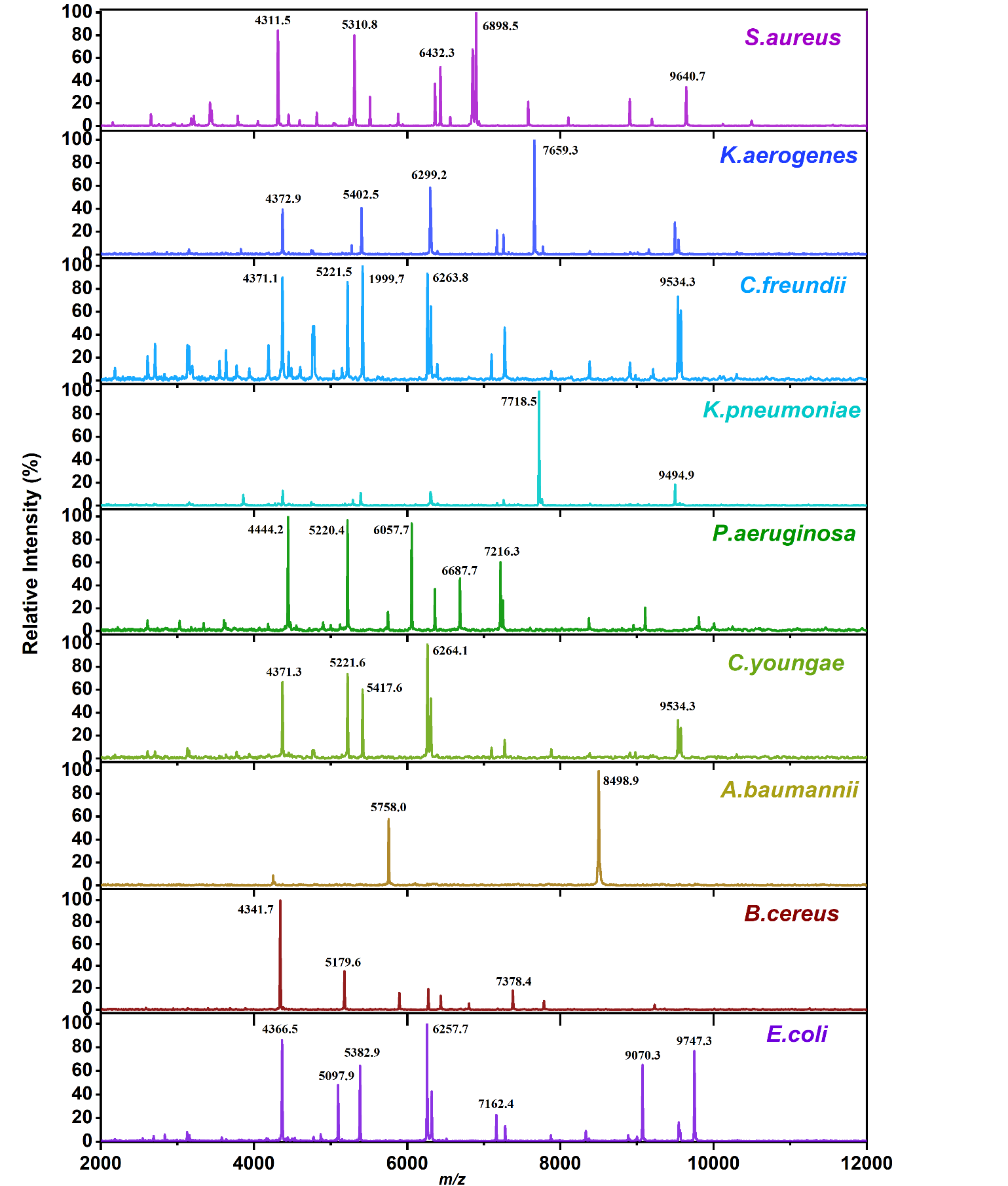


**Figure S1**. Mass spectra of single bacteria.


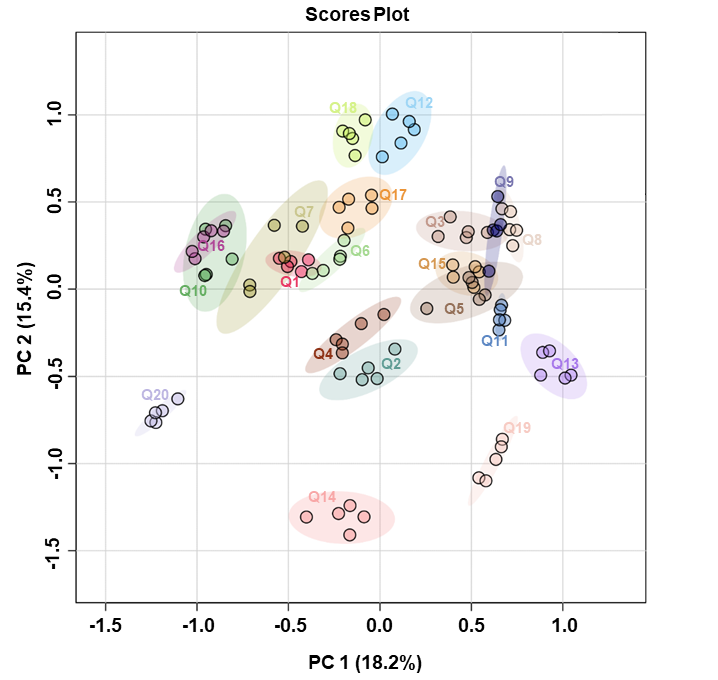
**Figure S2**. Principal component analysis (PCA)-based classification of actual model microbiota.


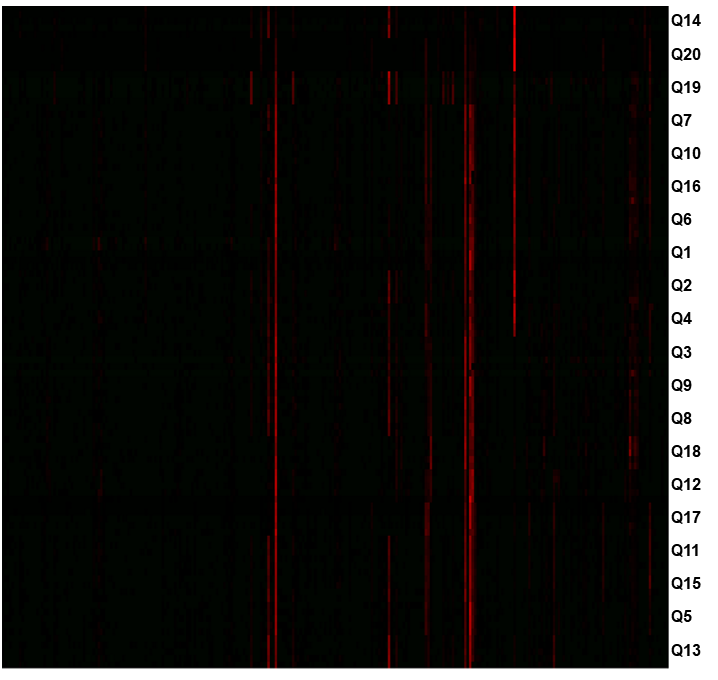
**Figure S3**. Heat map of actual model microbiota. A similar color of the same horizontal line means that the samples under this feature are similar, and a difference in color means that the samples under this feature are different.

**T****able S1**. Concentration and MALDI-TOF identification of single bacteria

| **Short name** | **Concentration**  **(×10^8^ CFU/mL)** | **Identification result** | | |
| --- | --- | --- | --- | --- |
|  |  | **Genus** | **Species** | **Score** |
| E.c | 9.1 | *Escherichia* | *coli* | 2.11 |
|  |  | *Escherichia* | *coli* | 2.13 |
|  |  | *Escherichia* | *coli* | 2.17 |
| B.c | 4.2 | *Bacillus* | *cereus* | 2.15 |
|  |  | *Bacillus* | *cereus* | 2.29 |
|  |  | *Bacillus* | *cereus* | 2.46 |
| S.a | 5.3 | *Staphylococcus* | *aureus* | 2.03 |
|  |  | *Staphylococcus* | *aureus* | 2.11 |
|  |  | *Staphylococcus* | *aureus* | 2.03 |
| C.y | 8.1 | *Citrobacter* | *youngae* | 2.32 |
|  |  | *Citrobacter* | *youngae* | 2.26 |
|  |  | *Citrobacter* | *youngae* | 2.05 |
| P.a | 7.5 | *Pseudomonas* | *aeruginosa* | 2.39 |
|  |  | *Pseudomonas* | *aeruginosa* | 2.33 |
|  |  | *Pseudomonas* | *aeruginosa* | 2.34 |
| K.a | 7.6 | *Klebsiella* | *aerogenes* | 2.39 |
|  |  | *Klebsiella* | *aerogenes* | 2.02 |
|  |  | *Klebsiella* | *aerogenes* | 2.23 |
| C.f | 8.3 | *Citrobacter* | *freundii* | 2.32 |
|  |  | *Citrobacter* | *freundii* | 2.32 |
|  |  | *Citrobacter* | *freundii* | 2.37 |
| A.b | 8.1 | *Acinetobacter* | *baumannii* | 2.22 |
|  |  | *Acinetobacter* | *baumannii* | 2.34 |
|  |  | *Acinetobacter* | *baumannii* | 2.30 |
| K.p | 6.8 | *Klebsiella* | *pneumoniae* | 2.15 |
|  |  | *Klebsiella* | *pneumoniae* | 2.07 |
|  |  | *Klebsiella* | *pneumoniae* | 2.35 |

**Table S2** Proportions in model microbiota

| **Microbiota number** | **Proportion (%)** | | | | | | | | | |
| --- | --- | --- | --- | --- | --- | --- | --- | --- | --- | --- |
|  | ***E.c*** | ***B.c*** | ***S.a*** | ***K.p*** | ***C.f*** | ***A.b*** | ***P.a*** | ***C.y*** | ***K.a*** |  |
| Q1 | 17.86 | 8.24 | 10.40 | 13.34 | 16.29 | 1.59 | 1.47 | 15.89 | 14.91 |  |
| Q2 | 1.82 | 8.41 | 10.61 | 13.61 | 16.61 | 16.21 | 15.01 | 16.21 | 1.52 |  |
| Q3 | 16.82 | 7.76 | 0.98 | 1.26 | 15.34 | 14.97 | 13.86 | 14.97 | 14.05 |  |
| Q4 | 18.11 | 8.36 | 10.55 | 13.54 | 1.65 | 16.12 | 14.93 | 1.61 | 15.13 |  |
| Q5 | 20.01 | 9.24 | 1.17 | 14.95 | 1.83 | 17.81 | 16.49 | 1.78 | 16.71 |  |
| Q6 | 18.82 | 0.87 | 10.96 | 1.41 | 17.17 | 16.75 | 1.55 | 16.75 | 15.72 |  |
| Q7 | 20.63 | 9.52 | 12.01 | 15.41 | 18.81 | 1.84 | 1.70 | 18.36 | 1.72 |  |
| Q8 | 1.98 | 9.15 | 1.15 | 1.48 | 18.07 | 17.64 | 16.33 | 17.64 | 16.55 |  |
| Q9 | 2.32 | 10.72 | 1.35 | 1.74 | 21.19 | 20.68 | 1.91 | 20.68 | 19.40 |  |
| Q10 | 22.56 | 1.04 | 13.14 | 16.86 | 20.58 | 2.01 | 1.86 | 20.08 | 1.88 |  |
| Q11 | 23.13 | 10.67 | 1.35 | 1.73 | 2.11 | 20.58 | 19.06 | 2.06 | 19.31 |  |
| Q12 | 2.22 | 1.03 | 1.29 | 16.60 | 20.26 | 1.98 | 18.31 | 19.77 | 18.55 |  |
| Q13 | 2.92 | 13.48 | 1.70 | 2.18 | 2.66 | 25.99 | 24.07 | 2.60 | 24.39 |  |
| Q14 | 3.13 | 14.44 | 18.22 | 2.34 | 2.85 | 27.84 | 25.78 | 2.78 | 2.61 |  |
| Q15 | 27.91 | 12.88 | 1.63 | 2.09 | 2.55 | 24.85 | 2.30 | 2.48 | 23.31 |  |
| Q16 | 27.68 | 1.28 | 16.12 | 20.69 | 2.53 | 2.46 | 2.28 | 24.64 | 2.31 |  |
| Q17 | 42.27 | 1.95 | 2.46 | 3.16 | 3.86 | 3.76 | 3.48 | 3.76 | 35.30 |  |
| Q18 | 14.00 | 6.46 | 8.15 | 10.46 | 12.77 | 12.46 | 11.54 | 12.46 | 11.69 |  |
| Q19 | 4.43 | 2.04 | 2.58 | 3.31 | 4.04 | 39.44 | 36.51 | 3.94 | 3.70 |  |
| Q20 | 46.76 | 2.16 | 27.2 | 3.49 | 4.27 | 4.16 | 3.85 | 4.16 | 3.91 |  |


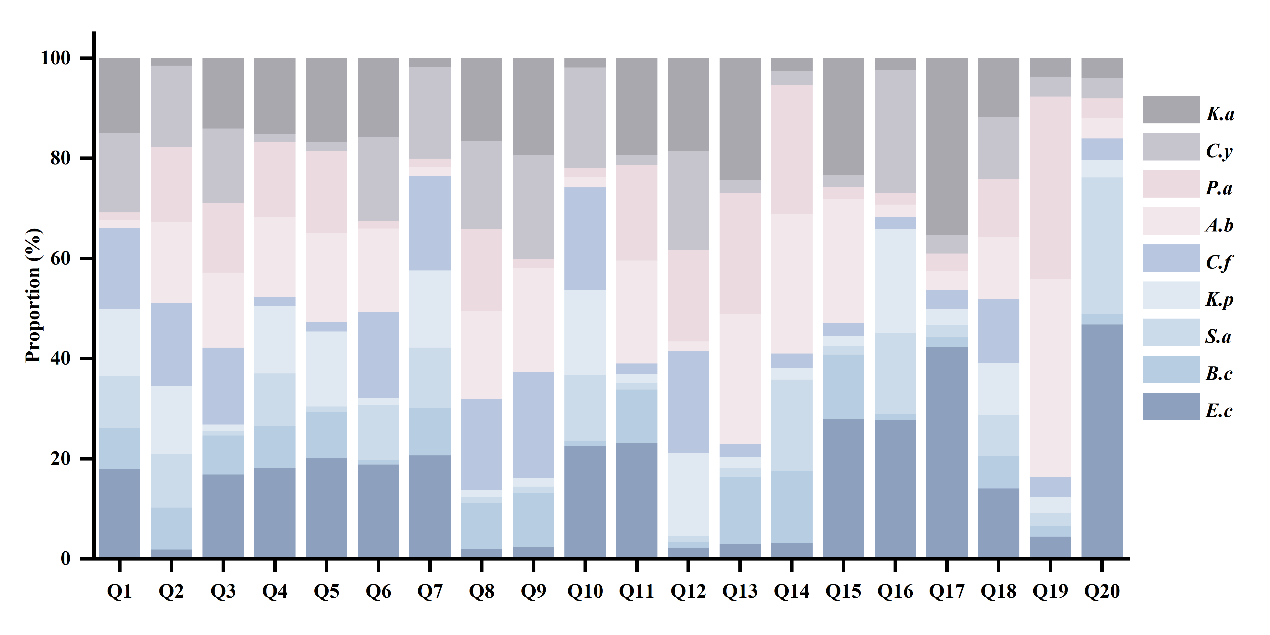


**Figure S4**. The proportion of model microbiota samples

**Table S3** Matching scores between the overlap spectra and actual spectra

| **Microbiota number** | **Score** | |
| --- | --- | --- |
| Q1 | 1.99 |  |
| Q2 | 1.75 |  |
| Q3 | 1.87 |  |
| Q4 | 1.61 |  |
| Q5 | 1.65 |  |
| Q6 | 2.02 |  |
| Q7 | 2.25 |  |
| Q8 | 1.64 |  |
| Q9 | 2.13 |  |
| Q10 | 2.19 |  |
| Q11 | 1.41 |  |
| Q12 | 2.07 |  |
| Q13 | 1.60 |  |
| Q14 | 1.65 |  |
| Q15 | 1.43 |  |
| Q16 | 2.13 |  |
| Q17 | 1.74 |  |
| Q18 | 1.81 |  |
| Q19 | 1.37 |  |
| Q20 | 1.81 |  |


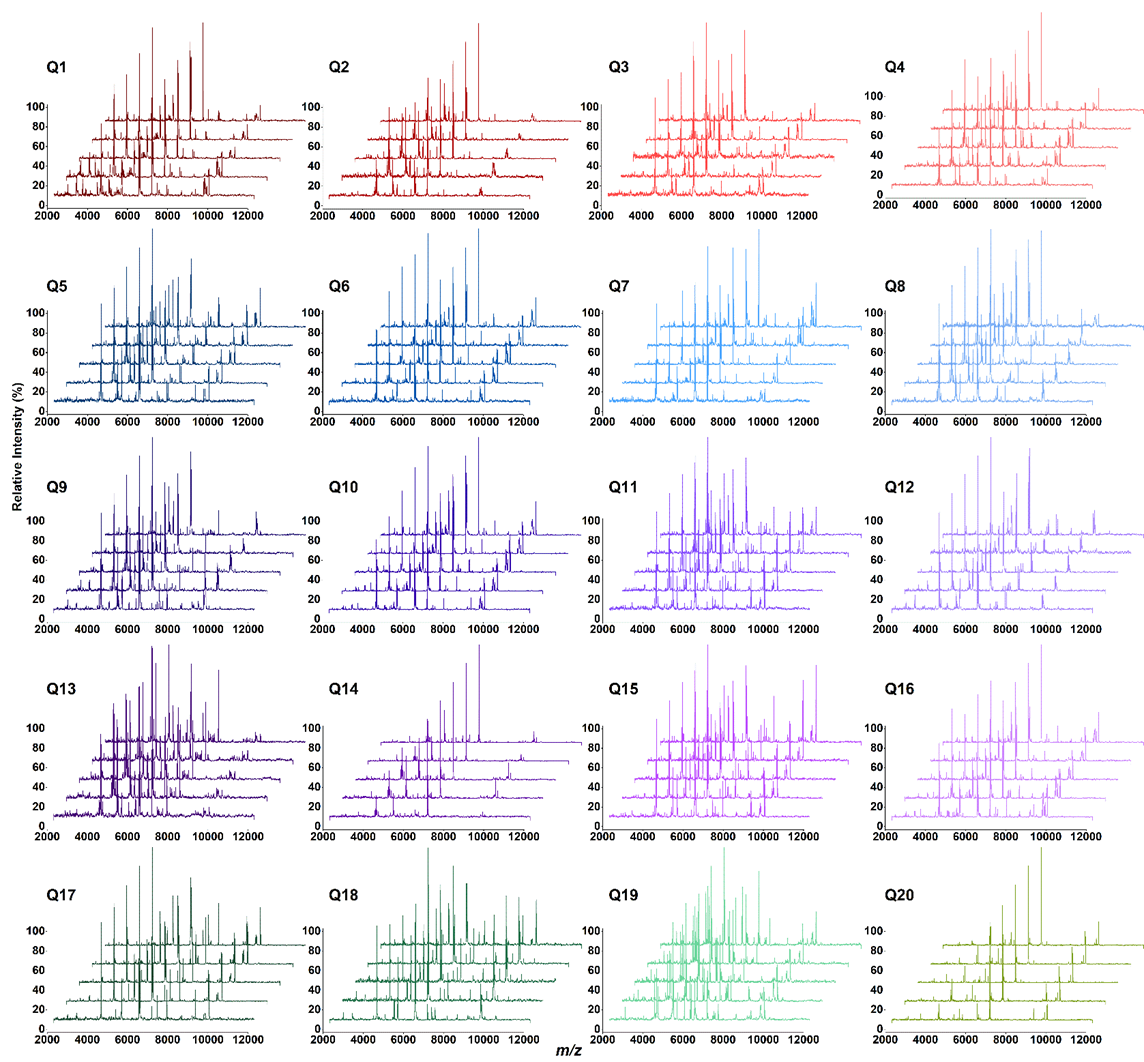


**Figure S5**. The reproducibility of actual collected model microbiota MS spectra. Each sample was repeated five times.


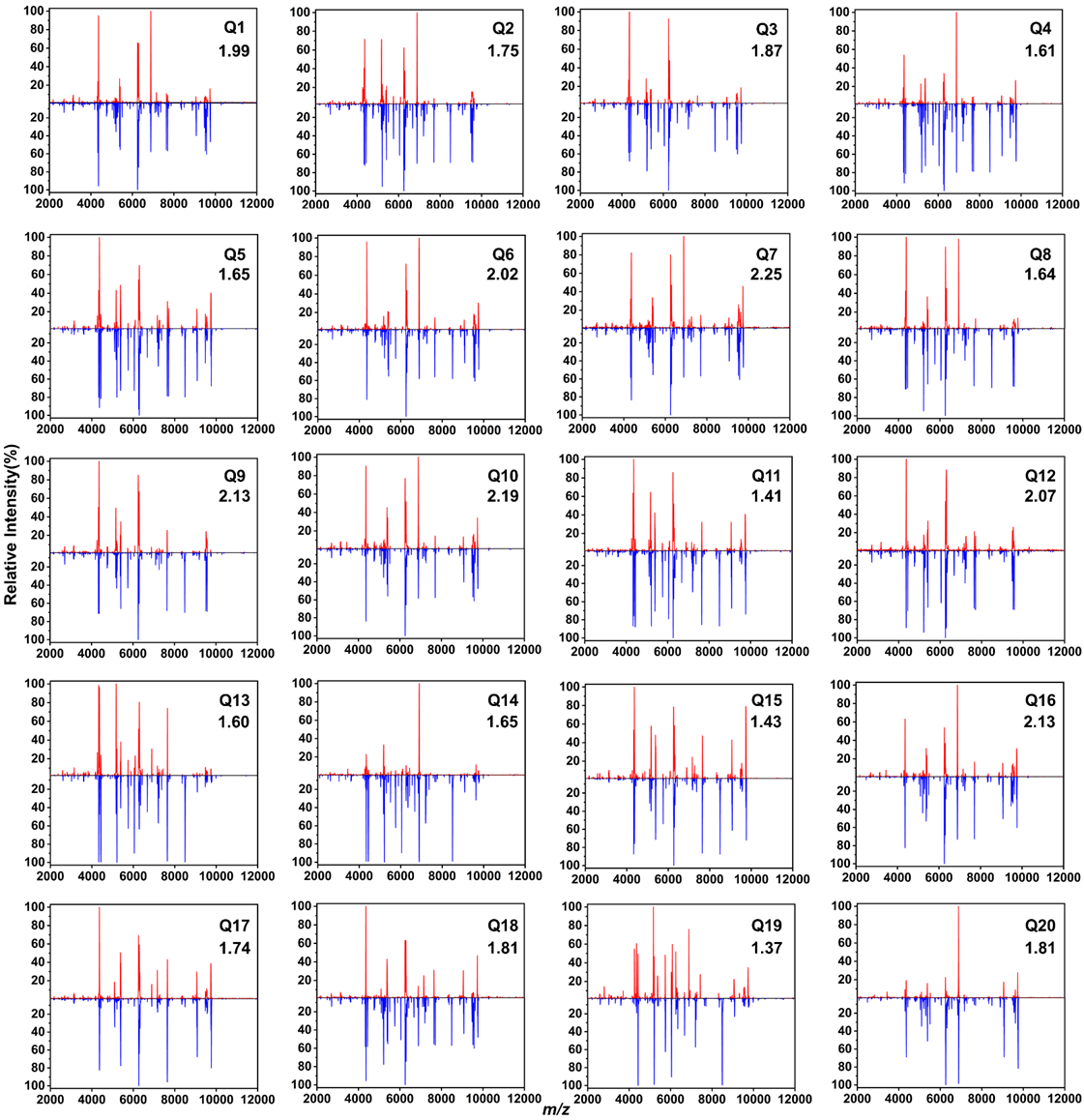


**Figure S6.** Actual collected MS spectra of 20 model microbiotas (red) and their corresponding overlap spectra (blue). The match score is shown in the upper right corner of each graph. A score >2.0 is considered a reliable result
